# Automated design prediction for definitive obturator prostheses: A case‐based reasoning study

**DOI:** 10.1111/jopr.13994

**Published:** 2025-01-04

**Authors:** Islam E. Ali, Mariko Hattori, Yuka Sumita, Noriyuki Wakabayashi

**Affiliations:** ^1^ Department of Advanced Prosthodontics, Graduate School of Medical and Dental Sciences Institute of Science Tokyo Tokyo Japan; ^2^ Department of Prosthodontics Faculty of Dentistry, Mansoura University Mansoura Egypt; ^3^ Department of Partial and Complete Denture School of Life Dentistry, The Nippon Dental University Tokyo Japan; ^4^ Institute of Science Tokyo Tokyo Japan

**Keywords:** artificial intelligence, clinical reasoning, denture design, maxillectomy, maxillofacial prosthodontics, obturator prosthesis

## Abstract

**Purpose:**

This study aims to evaluate the effectiveness of a case‐based reasoning (CBR) system in predicting the design of definitive obturator prostheses for maxillectomy patients.

**Materials and Methods:**

Data from 209 maxillectomy cases, including extraoral images of obturator prostheses and occlusal images of maxillectomy defects, were collected from Institute of Science Tokyo Hospital. These cases were organized into a structured database using Python's pandas library. The CBR system was designed to match new cases with similar historical cases based on specific attributes such as aramany class, abutment details, defect extension, and oronasal connection size. The system's performance was evaluated by clinicians who assessed the accuracy of prosthesis designs generated for 33 test cases.

**Results:**

A correlation analysis demonstrated a significant positive relationship (*ρ* = 0.84, *p* < 0.0001) between the CBR system's confidence scores and the number of correct prosthesis designs identified by clinicians. The median precision at five cases was 0.8, indicating that the system effectively retrieved relevant designs for new cases.

**Conclusions:**

The study shows that the developed CBR system effectively predicts the design of obturator prostheses for maxillectomy patients. Clinically, the system is expected to reduce clinician workload, simplify the design process, and enhance patient engagement by providing prompt insights into their final prosthetic design.

Artificial intelligence (AI) has revolutionized the field of dentistry over the past few years, enhancing the quality and efficiency of dental work.^1^ The more complex the treatment, the greater the benefit of AI integration to enhance patient care and help in clinical decision‐making.^2^ Such integration is expected to ultimately improve time, cost, and treatment efficiency. Maxillofacial prosthodontics plays a pivotal role in enhancing patients’ quality of life and self‐esteem by promptly addressing postsurgical defects. Demand for maxillofacial prosthodontics is forecast to increase over the next few decades due to several key factors.^3^ Super‐aging societies, marked by a growing older population, are prone to deterioration of oral health and heightened risk of cancer, particularly in the head and neck region.^4^ Traumatic injuries, such as those incurred in warfare, also contribute to the need for advanced maxillofacial rehabilitation.^3^, ^5^ Additionally, emerging health challenges such as COVID‐19‐induced mucormycosis^6^ and medication‐related osteonecrosis of the jaw^7^ further increase the demand for maxillofacial prosthetic interventions. However, maxillofacial prosthodontics is inherently complex, requiring consideration of various patient‐related factors during treatment planning. This, in turn, results in increased treatment duration and costs.^8^ Consequently, the incorporation of AI technology into the prosthodontic workflow has become imperative, given its potential to address the higher complexity associated with such cases and the growing number of cases that require prosthetic intervention.^2^

Previous studies explored the use of AI for image‐based classification of maxillectomy patients,^9^ predictions of the facial prosthesis color,^9^, ^10^ and the planning and production of naso‐alveolar molding devices for cleft palate patients.^11^, ^12^, ^13^, ^14^ Other general AI applications in prosthodontics include the classification of partially edentulous arches,^15^ designing removable partial dentures (RPDs),^15^, ^16^, ^17^ impression tray size selection,^18^ identifying the extension of the denture base,^19^ and other applications.^1^, ^2^, ^20^, ^21^

One interesting AI approach that has the potential to advance maxillofacial prosthodontics is case‐based reasoning (CBR), as delineated by Aamodt and Plaza.^22^ This methodology outlines a lifecycle consisting of four primary stages (retrieval, reuse, revise, and retain). During the retrieval phase, a novel problem is compared to prior cases stored in the case repository. Utilizing domain expertise, the system assesses the similarity between the new problem and past cases, gauging the suitability of previous solutions for the current problem. The most pertinent solutions are identified to address the current problem, possibly with modifications as needed. Subsequently, the chosen solution undergoes a revision process before being reused. Finally, both the new problem and its solution are preserved in the case library for future reference.^22^ While this framework provides a structured approach to CBR, it is important to recognize that CBR methodologies can be adapted to suit the specific characteristics of the problem at hand or to accommodate unique considerations.

The health sciences are a major area for the application of CBR. Within the medical context, where symptoms represent the problem, and diagnosis and treatment represent the solution, CBR is considered an appropriate method.^23^ Several fields of dentistry have explored the feasibility of using CBR as a decision support system.^24^, ^25^, ^26^, ^27^ In prosthodontics, this approach was used to design RPDs using the data of similarly treated cases to predict the design of a new case, with 96% overall accuracy.^17^ It was subsequently effective for mandibular and maxillary arches, with design correctness values of 100% and 75%, respectively.^16^ Maxillectomy defects are a common outcome of surgical treatments of oral cancers and other lesions involving the maxilla. Obturator prostheses of different designs are conventionally used for the treatment of such defects.^28^ However, the successful fabrication of these prostheses is not straightforward and is heavily influenced by multiple intraoral factors.^29^

The aim of this study is to investigate the effectiveness of a CBR system in predicting the design of definitive obturator prostheses for maxillectomy patients. This marks a step toward the automation of the prosthesis design process to facilitate standardized patient care and assist in clinical decision‐making. During the evaluation phase, clinicians assessed obturator designs generated by the CBR system. The null hypothesis asserts that there is no significant correlation between the confidence scores assigned by the CBR system to determine matching cases for a given new case and the number of correct obturator designs approved by clinicians among the system output.

## MATERIALS AND METHODS

### Database preparation

Data for the CBR system were retrospectively collected from dental records of patients treated at Institute of Science Tokyo Hospital between 1979 and 2024. The records represented extraoral images of both the polished and intaglio surfaces of definitive obturator prostheses that were delivered to a total of 209 dentulous maxillectomy patients. The inclusion criteria for these patients were as follows:

- Patients must have undergone maxillectomy with at least three remaining natural teeth.
- Definitive obturator prostheses must have been delivered to the patients.
- Available extraoral images of both the polished and intaglio surfaces of the obturator prostheses
- Radiographic records are available as a reference to the periodontal condition.
- Occlusal images of the maxillectomy defects are available as a reference to the oral condition of each case at the time of delivery of the definitive obturator prosthesis.
- Patients must represent different classes of Aramany^28^ (Figure S1), including those with and without surgical reconstruction.

The Aramany classification system is widely used to categorize maxillectomy defects based on the location and extent of the defect:

- Class I: hemi‐maxillectomy
- Class II: unilateral defect sparing the premaxilla on the defect side
- Class III: central palatal defect with intact dentition
- Class IV: the defect crosses the midline and involves both sides of the maxillae
- Class V: bilateral defect posterior to the remaining abutment teeth
- Class VI: the defect is anterior to the remaining abutment teeth ^28^

In this study, the CBR system was used to predict the design of definitive obturator prostheses, relying on matching new cases with stored cases of similar characteristics within the same Aramany class in the database. This ensures that the prosthesis design is tailored to the specific type of defect, enhancing the accuracy and effectiveness of the treatment.

Patient consent was obtained via an opt‐out method. A poster informing patients of the possibility of using patient data for research purposes was displayed in the clinic. For convenience and accessibility, information was also provided in digital format on the hospital's website. The study was approved by the ethical committee of the institution (approval no. #D2022004).

### Data organization and processing

A structured Microsoft Excel spreadsheet (Microsoft 365 MSO; Version 2403, Build 16.0.17425.20176, Microsoft Corp.) served as the primary data source, storing pertinent keywords (discussed in the next section) that describe the oral condition of various maxillectomy cases in the dataset. It also included the path of each case folder on the computer where image groups of the definitive obturator delivered to each case were separately stored. Compound figures showing the intaglio and polished surfaces were created for each case, to facilitate easy access by the code whenever a matching case was found. When the code is searching for a matching case in the database, the collected data are organized using the pandas library of Python (Version 3.11, Python Software Foundation), which transforms it into a structured pandas DataFrame. Each row within this DataFrame represented a distinct maxillectomy case, with individual columns specifying the keywords and attributes associated with that case. All coding tasks were performed in the PyCharm 2022.3.2 Integrated Development Environment (JetBrains, Prague, Czech Republic).

### Utilizing the CBR system for prosthetic design prediction

**FIGURE 1:**
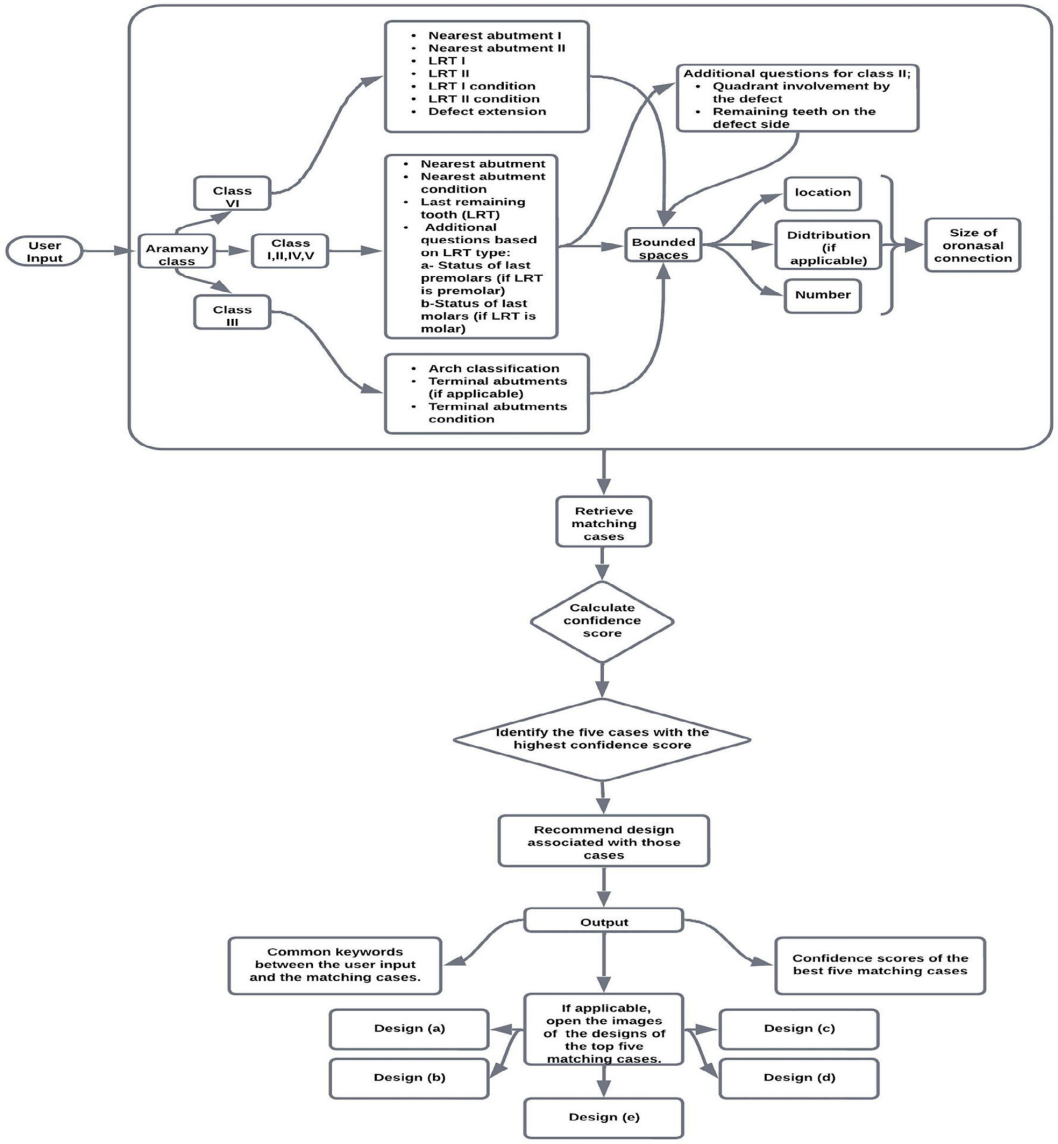
Flowchart of the CBR system illustrating the methodology for predicting the design of a new case. It shows the process of collecting user input, processing this input to identify top matching cases, and presenting these cases based on their confidence scores, including the opening of photos corresponding to the top five matching designs. CBR, case‐based reasoning.

The system operates on the principle that matching the oral condition of a new case, for which we wish to predict the design of a definitive obturator, with similar previously treated cases allows for the application of the same prosthetic designs. By systematically documenting key aspects of each new patient's oral condition through a structured questionnaire integrated into the CBR system (Figure 1), it can recommend prosthetic designs that have demonstrated efficacy in similar clinical scenarios. This questionnaire corresponds directly to how each case is described and organized within the Excel file database, which facilitates matching with previously treated cases. Based on this, the user is prompted to answer the following questionnaire to predict the obturator design for a new patient (Figure 1):

1. *Class identification*: The user identifies the Aramany class of the case (Figure S1), guiding subsequent questions and decisions.
2. *Abutment specification*: The user provides information regarding the nearest abutment to the defect and the last remaining tooth that may be involved in the prosthetic restoration, which is crucial for determining the structural foundation of the prosthesis (Figure S2). In Class VI cases, the user is prompted to answer these same questions twice, once for each side of the dental arch, further enhancing the precision of the prosthetic design process.
3. *Defect extension*: As the defect extension for most classes can be readily visualized upon determining the Aramany class, this item is only necessary for the user to decide in the case of Class II (whether it involves 1 or 2 quadrants) or in the case of Class VI (whether it is limited to the anterior region or extends for some distance onto the hard palate.
4. *Abutment condition*: The user evaluates the condition of the nearest abutment, considering factors such as periodontal health, integrity, and whether it is a lateral incisor (considered as a weak abutment that may require splinting to a nearby tooth) or remaining root suitable for use as an overdenture abutment (Figure S2). This assessment influences the selection of the optimal location for direct retainer placement, as well as the choice of material and design.
5. *Bounded spaces*: The user provides information regarding the number (single, double, triple, multiple, or no spaces), and location (anterior or posterior) of edentulous spaces in the remaining maxillary dentition (Figure S2). Additionally, for Class II, III, V, and VI cases, the user specifies the distribution of bounded spaces as unilateral or bilateral. These data guide the design and placement of prosthetic components.
6. *Oronasal connection size*: Determining the size of the oronasal connection helps assess the severity of the defect and guides decisions regarding prosthesis design and placement. Five distinct levels were identified: involvement of the entire defect, partial involvement of the defect, presence of a small fistulous connection (often blocked out before impression), complete surgical reconstruction, or a minimal defect without an oronasal connection (Figure S3).
7. *Remaining teeth on the defect side in Class II*: Since the remaining teeth have a bilateral distribution in Aramany class II, the user indicates whether the remaining teeth on the defect side are posterior only or both anterior and posterior. This follow‐up question arises when the nearest abutment to the defect is a premolar or molar and is skipped if it is an anterior tooth. It aims to determine the presence or absence of remaining anterior teeth on the defect side.
8. *Aramany class III* *cases*: When the defect primarily affects the hard palate and does not involve the teeth or alveolar ridge, a question on dental arch classification is utilized. To describe the existing situation, the user is prompted to classify the dental arch as a bilateral distal extension, unilateral distal extension, bounded saddle, or intact dental arch. In instances of bilateral or unilateral distal extension, the user is further prompted to identify the most posterior abutments. Additionally, questions related to the size of the oronasal connection and bounded spaces, and their distribution are included in the assessment process.
9. In cases where the last remaining tooth away from the defect is a premolar, the user is prompted to indicate whether both premolars are present. Similarly, if the last remaining tooth is a molar, the number of remaining molars is recorded. This information can help determine the design of the direct retainer and the placement of the components (Figure S2).

### Algorithm implementation for identifying top matching cases

The algorithm identifies the top matching cases through a systematic process (Figure 1). It begins by collecting user input regarding the characteristics of the prosthodontic case under consideration as shown in Figure 1. The questions posed to the system are dynamically customized based on the user's response regarding the Aramany class of the case. This customization is crucial for accommodating the anatomical and design disparities inherent across different Aramany classes. Moreover, the user's answers to certain questions dictate the subsequent questions that will appear, ensuring a logical flow and relevance in the inquiry process. For example, if the user answers “no spaces” to the question about the number of bounded spaces, the code will skip the question asking about their location. Similarly, if the answer is “double spaces,” the user will receive follow‐up questions about their location (anterior/posterior) (Figure S2) and distribution (unilateral/bilateral; for all classes except Class I and IV since the remaining teeth are on one side of the dental arch).

**FIGURE 2:**
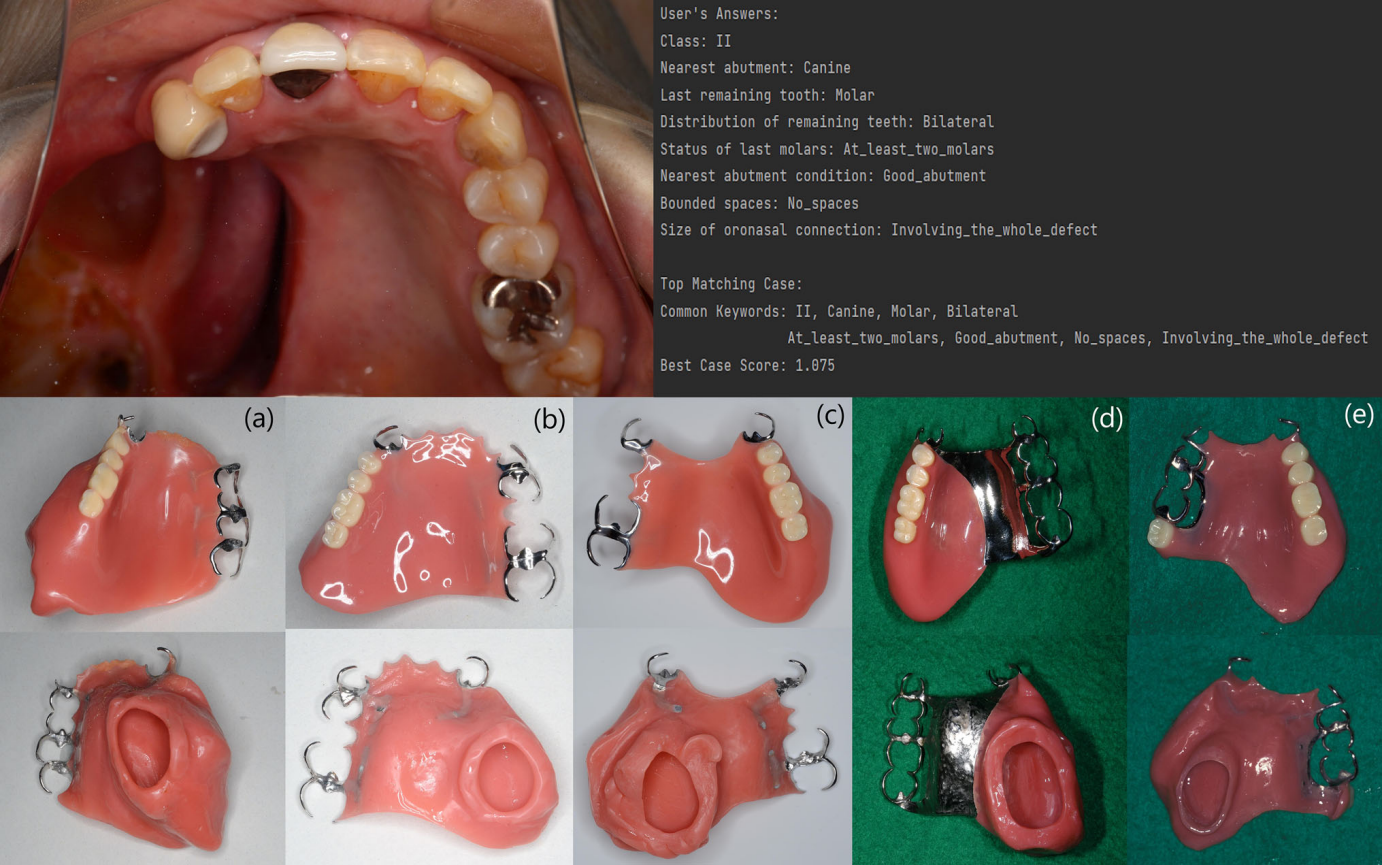
This figure presents the output generated by the model for a new case. Designs (a), (b), (c), and (d) received a confidence score of 1, indicating a high level of alignment with the user's input. However, design (e) received a slightly lower confidence score of 0.95. This was attributed to a discrepancy in the number of remaining molars.

Once the user inputs are gathered, the algorithm compares them with the keywords of each case in the database. The algorithm assigns a confidence score to each case based on the number and importance of matching keywords. Cases with higher confidence scores are considered to be more closely aligned with the user's inputs. The top matching cases are then selected based on their confidence scores. The algorithm presents these top cases to the user, along with common keywords shared between the user's inputs and the selected cases (Figure 2). Finally, it shows the predicted designs by opening the extraoral photos of the definitive obturator prostheses delivered to the identified matchings (Figure 2).

## Evaluation

**FIGURE 3:**
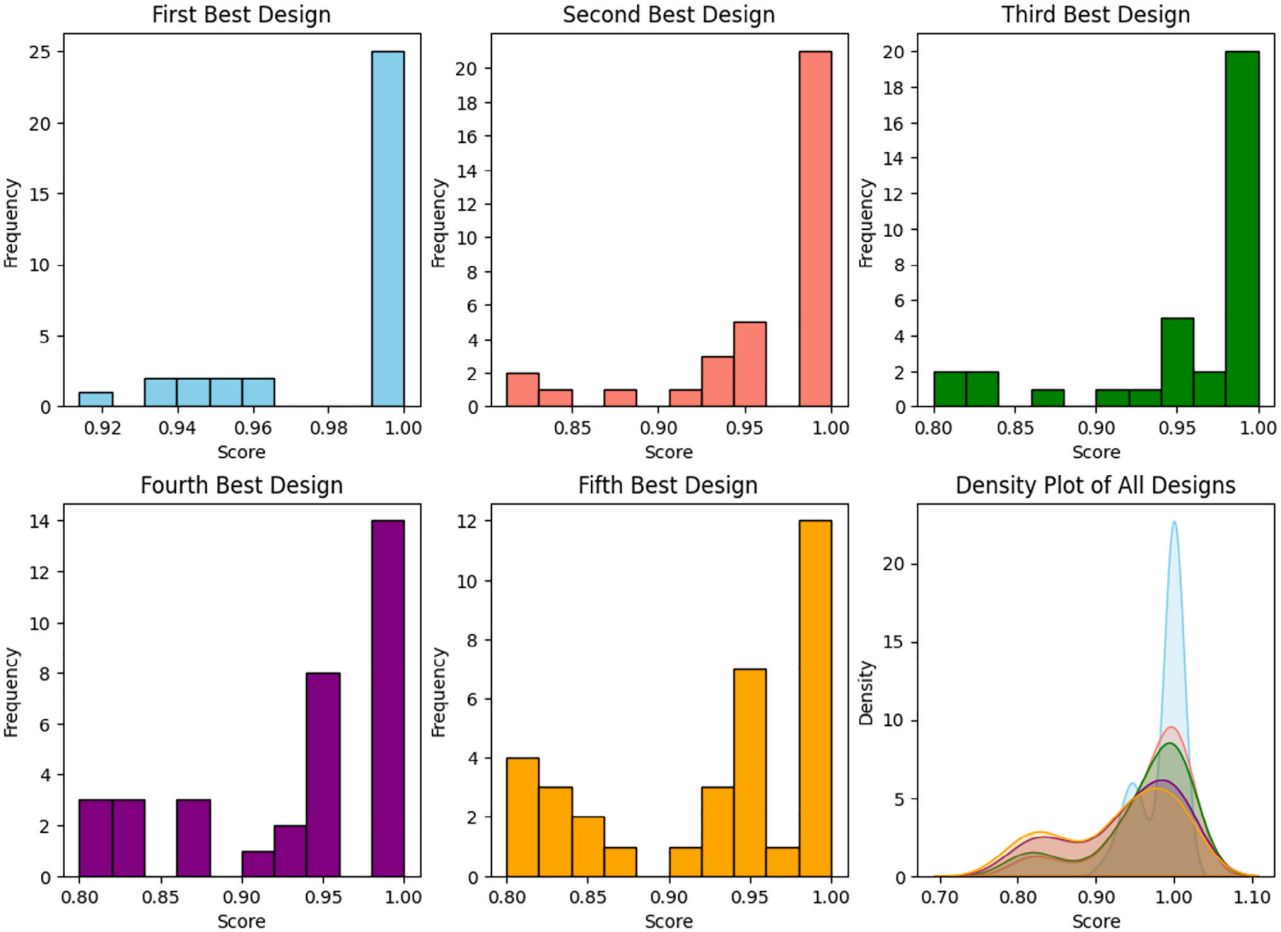
Histograms and density plot of the confidence scores associated with the five best design outputs.

Definitive obturator work authorizations (*n* = 33) were selected for assessment using a random sampling method without replacement from cases not included in the CBR DataFrame. A set of obturator designs was compiled for each patient, with their similarity values calculated by a confidence score in descending order (Figure 3). In this study, the top 5 matching cases were chosen and were evaluated for design correctness by two professionals with over 20 years of experience. The evaluations were based on the design criteria and philosophy taught at the university and some common references.^28^, ^29^ A design is deemed matching for a given new case if it satisfies the following criteria:

1. It corresponds to the same Aramany class.
2. The corresponding matching case has a confidence score above 90%, ensuring adequate similarity in the oral condition between the new case and the matching case.
3. Direct and indirect retainers can be applied in the new case following similar arrangements and locations.
4. The major connector design or denture base extension provides the necessary stabilization and rigidity to withstand tortional stress according to the defect size and condition of the remaining teeth.
5. The bulb portion design is appropriate for the size of the defect, which includes solid or hollow types (either open or closed).
6. It fulfills the required amount of retention, support, and stability needed for a maxillofacial prosthesis while acknowledging variations in complexity among cases.

The confidence scores of the top matches were recorded and the number of correct designs identified by the two professionals among the design outputs were counted for each test sample and used for further analysis. Precision, a fundamental metric for evaluating model effectiveness,^17^ was also calculated as the fraction of retrieved cases that matched the final design.

### Statistical analysis

The statistical analysis aimed to investigate the relationship between the averages of the five confidence scores of the top five cases and the number of correct designs identified by clinicians. This investigation sought to assess the efficiency of the matching keywords in retrieving accurate designs, focusing on two dependent factors: the confidence scores and the number of correct designs. First, the normality of the data distributions was assessed using Shapiro–Wilk tests, which revealed that the two datasets were not normally distributed (*p* < 0.05). Due to the non‐normal distribution of the data, a nonparametric correlation test was conducted to assess the association between the two factors. Specifically, Spearman's rank correlation coefficient (*ρ*) was employed to evaluate the strength and direction of the relationship. Precision at the top five matchings was also calculated. The analyses were performed using JMP version 16 (JMP Statistical Discovery LLC, Cary, NC, USA). An alpha level (*p*) of <0.05 was considered statistically significant.

## RESULTS

For each test patient, five designs were predicted in descending order of recommendation based on their confidence scores. The confidence scores across all patients ranged from 0.8 to 1 for the best five matching designs, with a median of 1. The clustering of confidence scores around 1, as depicted in Figure 3, indicates a high degree of agreement in the oral conditions within the constraints of the questionnaire. This clustering is attributed to the inclusion of patients with similar oral conditions but different designs in the database, allowing a confidence score of 1 even for the fifth‐best design. This flexibility demonstrates that the system, based on matching oral conditions, can recommend multiple suitable designs for similar cases. Furthermore, certain keywords were given higher weight in the confidence score calculation, which led to slightly inflated scores and a higher frequency of confidence scores reaching 1. This weighting prioritizes clinically relevant cases in the matching process, thereby enhancing the accuracy of the recommended designs.

**FIGURE 4:**
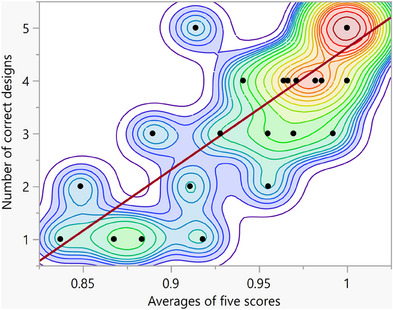
A correlation analysis shows a strong positive correlation between the averages of the five scores associated with each case and the number of correct designs determined by clinicians. A quantile density plot depicting non‐parametric correlation is displayed in the background.

The correlation analysis demonstrated a significant positive relationship (*ρ* = 0.84, *p* < 0.0001) between the averages of the five confidence scores of the top five matching designs and the number of correct designs identified among these top matches, as judged by the two professionals (Figure 4; Table 1). This finding suggests that higher confidence scores are reliably associated with a greater number of correct prosthetic designs, highlighting the efficacy of the questionnaire in retrieving relevant designs. It also indicates that using the Aramany classification as a guiding framework effectively aids in the accurate matching of prosthetic designs to new patients.

**TABLE 1:** Summary statistics and correlation results of the confidence scores and corresponding correct design counts.

	Minimum	Maximum	Median	Spearman's *ρ*	*p*‐value
Average of the five confidence scores per case	0.837	1	0.982	0.84	<0.0001*
Number of correct designs per case	1	5	4		

* *p* < 0.05 is considered statistically significant.

**FIGURE 5:**
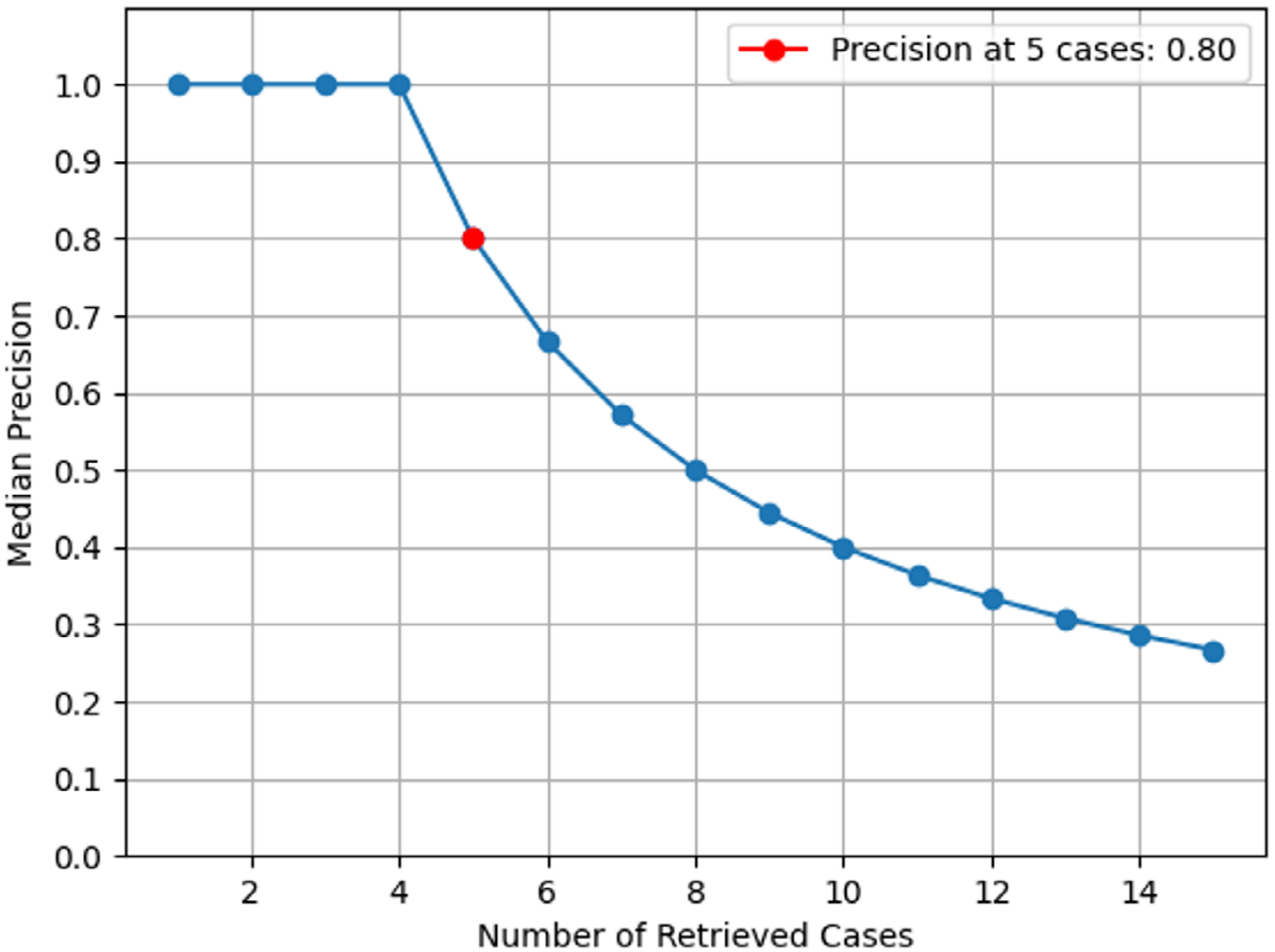
Precision at the top 1–15 cases retrieved by the model.

The precision at the top 1–15 is shown in Figure 5. The median precision at the five‐case threshold was 0.8. Beyond this threshold, the likelihood of finding a relevant design decreases, leading to lower precision values. The current high precision at this threshold can be attributed to factors such as a relatively small test sample size and the predominance of Class I and II cases in the database used for matching which covers a broader range of patient variations and thereby increases the likelihood of finding a matching case. Additionally, extensive defects such as Class I and IV involve significant tissue loss, where remaining healthy tissue is relatively uniform among cases covering half or less than half of the maxilla, respectively. This similarity limits variations in oral conditions among cases, resulting in higher confidence scores, thus facilitating easier matching and increasing precision.

## DISCUSSION

This study investigated the effectiveness of a CBR system in facilitating the design of definitive obturator prostheses for maxillectomy patients. Using data from a comprehensive repository of clinical cases, the analysis revealed several important insights into the utility and potential of AI‐driven approaches in maxillofacial prosthodontics. First, we observed a significant positive relationship between the confidence scores assigned by the CBR system and the accuracy of designs determined by clinicians. Therefore, the null hypothesis was rejected. This finding highlights the reliability of the model in retrieving precise designs that align with clinical expectations. Furthermore, achieving a precision of 0.8 for the top five matching cases serves as a strong indicator of the system's effectiveness in recommending relevant designs.

Chen et al.^17^ developed a CBR software program to automate the design process of RPDs. The RPD designs for 104 patients were compared with those made by professionals, yielding a mean precision average of 0.61. In a subsequent evaluation,^16^ the same software program was further assessed. Clinician and software‐generated designs for 112 treatments were compared by two prosthodontic department clinicians. The investigation revealed a 100% correctness rate for mandibular designs and 75% for maxillary arches. Additionally, the precision achieved was 100% for the mandible and 75% for the maxilla. While the study by Chen et al.^17^ was based on the philosophy of a single dental school, the system developed in the present study solely relies on describing the oral condition without defining rigid rules for designing, making it more versatile and adaptable for use in various settings. The keywords were designed to consider various factors affecting the design's complexity and component placement. Additionally, the system output in previous studies was either based on textual descriptions of framework components and their location^17^ or 2‐dimensional diagrams of the design.^16^ In contrast, the system developed in the present study provides photos depicting the actual prosthesis delivered for cases with similar oral conditions.

A number of benefits of applying CBR in the medical domain have already been identified.^23^ The advantages of the developed system include the simplicity of the algorithm, which enhances the speed of its execution, and the standardized preparation of keywords for all cases, allowing for easy and straightforward matching. Additionally, since the questions are designed to describe the existing oral condition, there is no need to take intraoral photos for an AI‐based design system that depends on feature extraction from training images.^30^ This is particularly helpful in case of trismus or microstomia where it is often challenging to take intraoral photos. Additionally, a set of standard cases is sufficient to find a matching case.^17^, ^25^, ^26^, ^27^ The greater the variety the higher the chance of finding a matching case. However, the dataset will still be much smaller than that needed for a system based on training and feature extraction and, therefore, does not require high computational resources.^30^

Another advantage is that the patient can immediately see the final design after the attending clinician completes the questions about the case, facilitating a better understanding and providing a more informative explanation. Additionally, displaying the intaglio surface of the predicted design can assist maxillofacial prosthodontists and dental technicians in visualizing the anticipated structure based on the defect size. Although the intaglio surface of the obturator bulb is ultimately determined by the impression of the defect, this visualization aids in choosing the appropriate bulb portion design, such as solid or hollow (open or closed). The developed CBR system can offer assistance and guidance to dentists lacking extensive experience in maxillofacial prosthodontics.^1^, ^30^ Considering the increasing demand for maxillofacial prosthodontics, there is a shortage of maxillofacial prosthodontists to meet these demands.^3^, ^31^ Consequently, general dentists will be increasingly tasked with treating patients with head and neck defects, making the support provided by the CBR system even more critical.

An added advantage of the system lies in its capability to generate the top five matching cases (or more if needed) rather than a singular output. This design choice introduces greater flexibility and accommodates variation between cases, thereby enhancing the clinician's ability to discern and select the most suitable design for a given case. As an educational tool, a dental student will be able to try different inputs and view the corresponding design output, which allows for judgment of their own designs and a better understanding of how the design is affected by the existing oral condition.^32^

However, there are notable disadvantages that need to be addressed. The system's performance is highly dependent on the accuracy of the input provided by the user. Inaccurate or incomplete data can lead to suboptimal design outputs. Additionally, a limited dataset may reduce the chances of finding closely matching cases, which could impact the precision of the design recommendations. Likewise, the design output of this system is exclusively focused on conventional obturator design and does not incorporate implant‐based prosthodontics as a viable option. This decision stemmed from the prevalent preference among the majority of patients, who typically select options that are covered by insurance, as well as the difficulty of planning implant‐based prosthodontics for maxillectomy patients relative to patients without defects.^8^, ^33^ However, for future iterations of the system, the inclusion of dental implants in the design process is warranted. While the system relies on the commonly used and widely known Aramany classification,^28^ it acknowledges the inherent limitations of such classification systems. These limitations include overlooking the vertical extension of defects, defects confined to the vestibule or soft palate, those bounded by teeth at both ends and multiple discontinuous defects. Additionally, some defects are less commonly encountered in our clinic, such as Aramany class III, V, and VI which limit the options that the system can use for matching. Future enhancement of the system should incorporate additional details pertaining to the opposing arch condition. Factors such as inter‐arch space, tongue movement, particularly for mandibulectomy patients, and the characteristics of the opposing occlusion merit inclusion to enhance the system's comprehensiveness and effectiveness.

## CONCLUSIONS

This study demonstrates that the developed CBR system can successfully predict the design of definitive obturator prostheses for maxillectomy patients. This system offers a practical approach to prosthetic design, aiding clinicians in decision‐making and treatment planning. The ability to generate multiple design predictions adds flexibility to the decision‐making process, allowing clinicians to make informed choices based on patient needs. Clinically, the system is expected to reduce clinician workload, simplify the design process, and enhance patient engagement by providing prompt insights into their final prosthetic design. Moreover, it has the potential to support clinicians with varying levels of expertise in maxillofacial prosthodontics, improving overall patient care.

## CONFLICT OF INTEREST STATEMENT

The authors declare no conflicts of interest.

## Supporting information



Supporting information

Supporting information

Supporting information
