# Supplementary figures and images for: Automated design prediction for definitive obturator prostheses: A case‐based reasoning study
Source: J Prosthodont. 2025 Jan 4;34(5):490–9. doi: 10.1111/jopr.13994 (PMC12147418; doi:10.1111/jopr.13994)

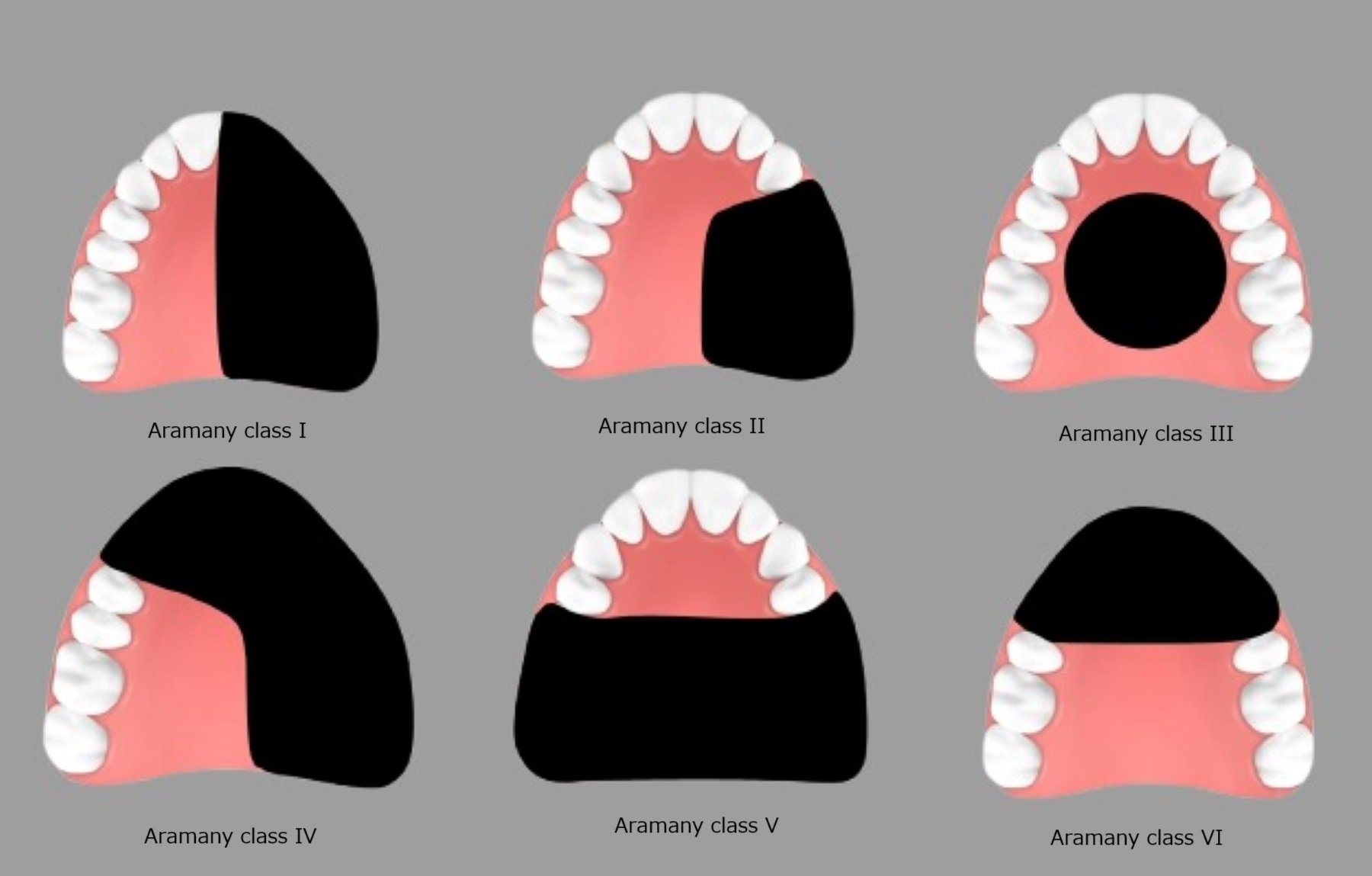

Supplement: Supplementary file 1 — Supporting information [file JOPR-34-490-s002.tiff]

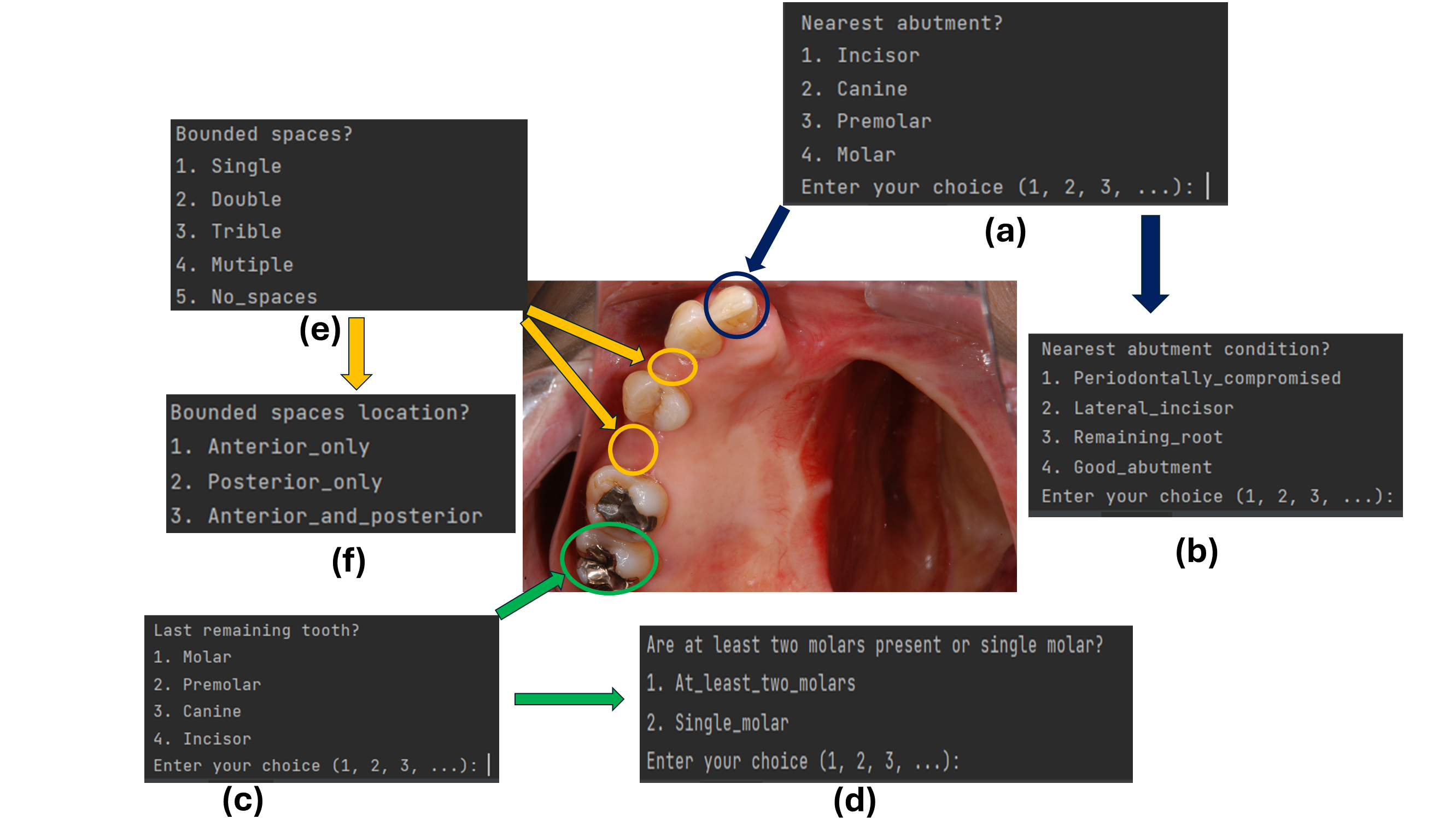

Supplement: Supplementary file 2 — Supporting information [file JOPR-34-490-s001.tif]

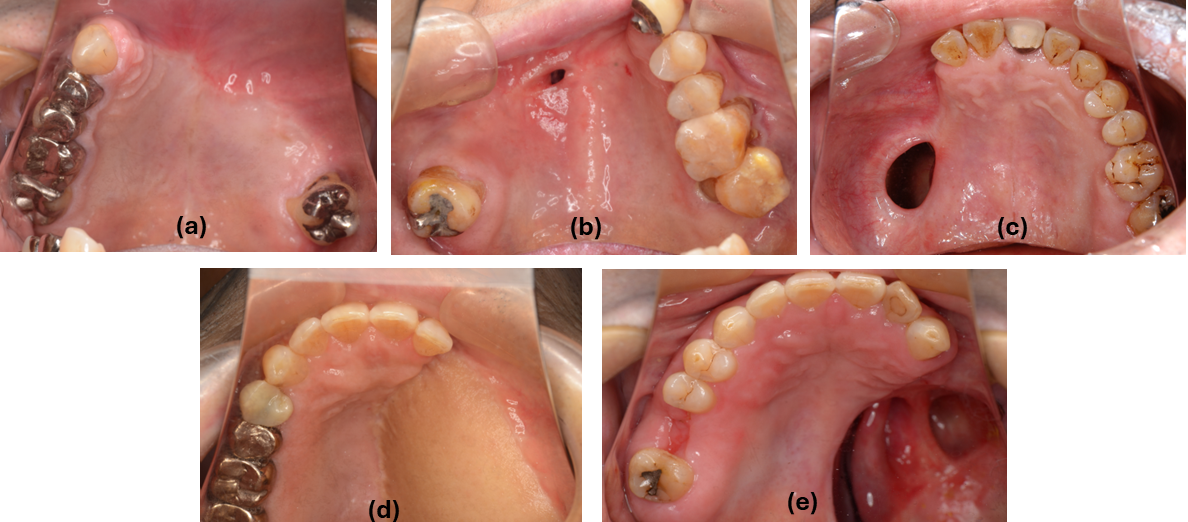

Supplement: Supplementary file 3 — Supporting information [file JOPR-34-490-s003.tif]
